# Severe COVID-19 in Third Trimester Pregnancy: Multidisciplinary Approach

**DOI:** 10.1155/2020/8889487

**Published:** 2020-10-14

**Authors:** Jerald Pelayo, Gabriella Pugliese, Grace Salacup, Eduardo Quintero, Adeeb Khalifeh, David Jaspan, Bhavna Sharma

**Affiliations:** ^1^Department of Medicine, Einstein Medical Center Philadelphia, Philadelphia, PA, USA; ^2^Department of Obstetrics and Gynecology, Einstein Medical Center Philadelphia, Philadelphia, PA, USA; ^3^Division of Maternal Fetal Medicine, Einstein Medical Center Philadelphia, Philadelphia, PA, USA; ^4^Division of Pulmonary and Critical Care and Sleep Medicine, Einstein Medical Center Philadelphia, Philadelphia, PA, USA; ^5^Sidney Kimmel College of Thomas Jefferson University, Philadelphia, PA, USA

## Abstract

The rapidly expanding cases of the coronavirus disease 2019 (COVID-19) caused by the severe acute respiratory syndrome coronavirus 2 (SARS-CoV-2) have exposed vulnerable populations, including pregnant women to an unprecedented public health crisis. Recent data show that pregnancy in COVID-19 patients is associated with increased hospitalization, admission of the intensive care unit, and intubation. However, very few resources exist to guide the multidisciplinary team in managing critically ill pregnant women with COVID-19. We report our experience with managing a morbidly obese pregnant woman at 36 weeks' gestation with history of asthma and malignancy who presented with persistent respiratory symptoms at an outside hospital after being tested positive for SARS-CoV-2 polymerase chain reaction (PCR). Early in the course of the hospitalization, patient received remdesivir, convalescent plasma, bronchodilator, systemic steroids, and IV heparin for COVID-19 and concomitant asthma exacerbation and pulmonary embolism. Due to increasing oxygen requirements, she was eventually intubated and transferred to our institution for higher level of care. Respiratory acidosis, severe hypoxemia, and vent asynchrony were managed with vent setting adjustment and paralytics. After 12 hours from spontaneous rupture of her membranes and with stabilization of maternal status, patient underwent a term cesarean delivery for nonreassuring fetal heart tracing. The neonate was discharged on the 2^nd^ day of life, while the patient was extubated on the 6^th^ postpartum day and was discharged to acute inpatient rehabilitation facility on the 19^th^ hospital day. This report highlights the disease progression of COVID-19 in a pregnant woman, the clinical challenges in the critical care aspect of patient management, and the proposed multidisciplinary strategies utilizing an algorithmic approach to optimize maternal and neonatal outcomes.

## 1. Introduction

Deemed as one of the most profound public health threats over the past decades, COVID-19 has now been reported from all continents except for Antartica, affecting 7,124,043 individuals in 118 countries with the recorded mortality rate of 5.7% as of June 8, 2010 [1]. It has exposed vulnerable populations, including the pregnant patients, to an unparalleled global health crisis.

Outcomes from multiple studies demonstrated a significant risk of morbidity and mortality among pregnant women infected by influenza, severe acute respiratory syndrome (SARS), and Middle East respiratory syndrome (MERS) compared to nonpregnant women [2–4]. Based on the previous limited data, this particular patient population is not at higher risk for developing severe disease due to COVID-19 compared to the general population [1, 5, 6]. However, the most recent data from the Centers for Disease Control and Prevention (CDC) showed the pregnant women with COVID-19 are more likely to be hospitalized, admitted to the intensive care unit, and intubated [7].

Data on the effect of COVID-19 in pregnancy in large retrospective study of 119 pregnant women and systematic review of 109 pregnancies showed a 2.7% to 6.9% incidence rate of severe infection requiring ICU admission with no maternal deaths [8, 9]. However, there has been no one-size-fits-all approach in managing critically ill pregnant women with COVID-19 given unique physiologic maternal adaptations and important management and ethical considerations. Furthermore, few contemporary resources are available to guide the multidisciplinary team through decisions regarding intensive care strategies, optimal maternal-fetal surveillance, and route as well as timing of delivery.

We present a case of a woman at 36 weeks' gestation who had a rapid clinical deterioration from COVID-19-related ARDS to highlight clinical challenges and potential strategies through multidisciplinary team approach to optimize maternal and neonatal outcomes.

## 2. Case Presentation

**Figure 1:**
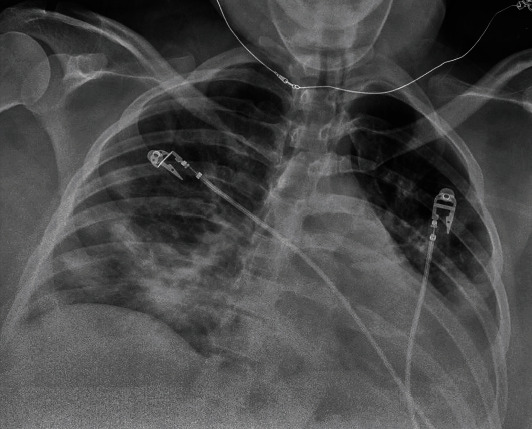
Chest radiograph on admission at the outside hospital showing interval worsening of hazy airspace opacities in the bilateral mid to lower lung fields.

**Figure 2:**
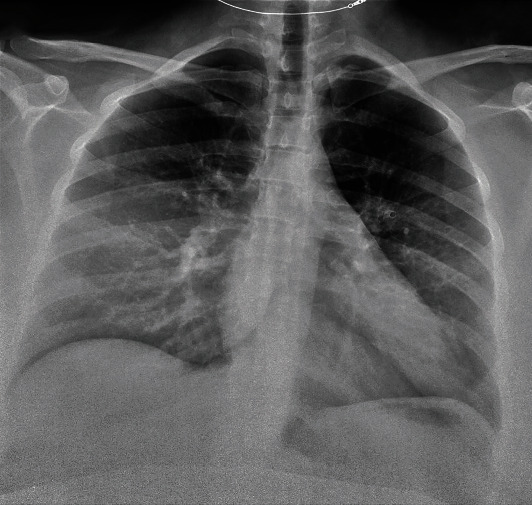
Chest radiograph on prior ED visit showing haziness overlying the right mid and lower lung fields.

**Figure 3:**
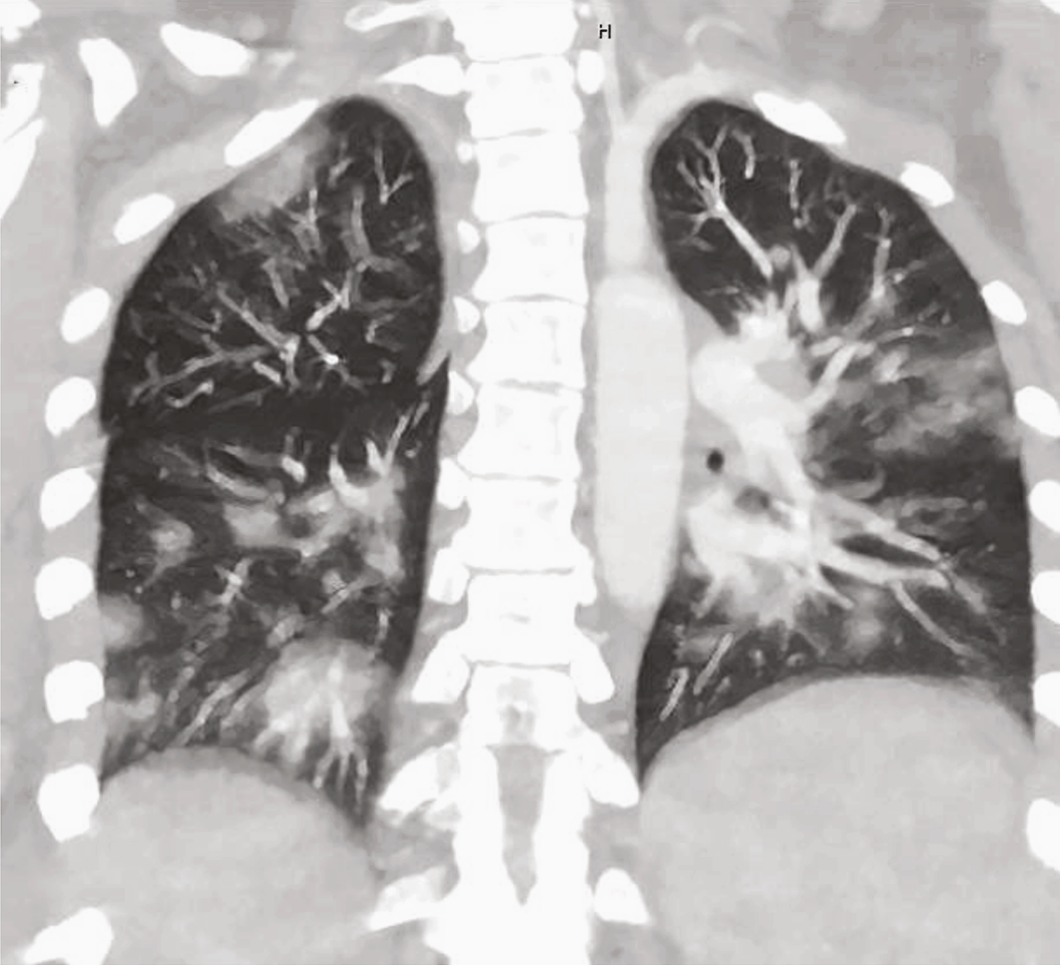
CT Pulmonary angiography (coronal section) showing extensive bilateral ground-glass and consolidative opacities with peripheral distribution and small bilateral pleural effusion.

**Figure 4:**
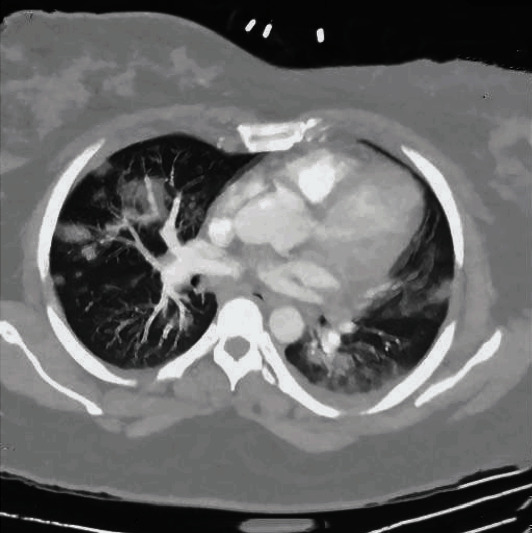
CT Pulmonary angiography (axial section) showing extensive bilateral ground-glass and consolidative opacities with peripheral distribution, small bilateral pleural effusion, and multiple bilateral intraluminal filling defects.

Patient is a 35-year-old G4P1-1-1-2 with a past medical history notable for mild intermittent asthma, hepatitis C, gastric carcinoid tumor, ileal malignant carcinoma S/P hemicolectomy, morbid obesity (body mass index of 51 kg/m^2^), abdominal hernia s/p mesh repair, bipolar, and anxiety disorder, with a confirmed SARS-CoV2 PCR after a recent known exposure to a positive household contact. She was admitted to a tertiary care hospital after failing self-quarantine measures at home, with persistent nonproductive cough, hemoptysis and worsening shortness of breath without significant response to frequent albuterol nebulization at home. At the time of admission, age of gestation by the last menstrual period (LMP) was 36 2/7 weeks. At presentation, she was normotensive, tachycardic, and tachypneic but not hypoxic on room air. She had diffuse wheezing and rhonchi on lung auscultation. Significant laboratory findings include thrombocytopenia (98,000/uL), normocytic anemia with normal haptoglobin level (145 mg/ml), elevated LDH of 264 IU/L with mild transaminitis (AST 53 IU/L, ALT 53 IU/L) without schistocytes on peripheral blood smear and normal bilirubin levels, elevated ferritin (140 ng/mL), and D-dimer (2810 ng/mL). The nonstress test was reactive with reassuring fetal heart tracing. Chest radiograph (Figure 1) showed worsening bilateral pulmonary opacities compared to the previous (Figure 2). CT pulmonary angiography demonstrated extensive bilateral ground-glass opacities with peripheral distribution, small bilateral pleural effusion, and multiple bilateral intraluminal filling defects of the pulmonary arteries suggestive of pulmonary embolism (Figures 3 and 4).

The patient was admitted to the general medical floor where she received convalescent plasma transfusion, in addition to bronchodilators and intravenous methylprednisolone. She was started on a 10-day course of remdesivir treatment. Over the next four days, she had increasing oxygen requirement from 4 L to 12 L via nasal cannula to keep her oxygen saturation above 92%. On the 5^th^ day of admission, patient was noted to be agitated and hypoxic to 88% on 40 L of oxygen via a combination of nasal cannula with nonrebreather mask on it. During this time, fetal heart tracing was reassuring. Tocogram showed contractions every 4 to 6 minutes. She was then transferred to the medical intensive care unit (MICU) to attempt high flow nasal cannula (HFNC). After persistent hypoxia on HFNC, the decision was made to intubate the patient. Mechanical ventilation with assist control/volume control settings includes tidal volume (TV) of 500 ml (7 ml/kg), respiratory rate (RR) of 25, positive end expiratory pressure (PEEP) of 14, and fraction of inspired oxygen (FiO2) of 100%. Heparin, *cis*-atracurium, fentanyl, and propofol drips were initiated and continued. She was then transferred to our institution via air ambulance for higher level of care.

**Figure 5:**
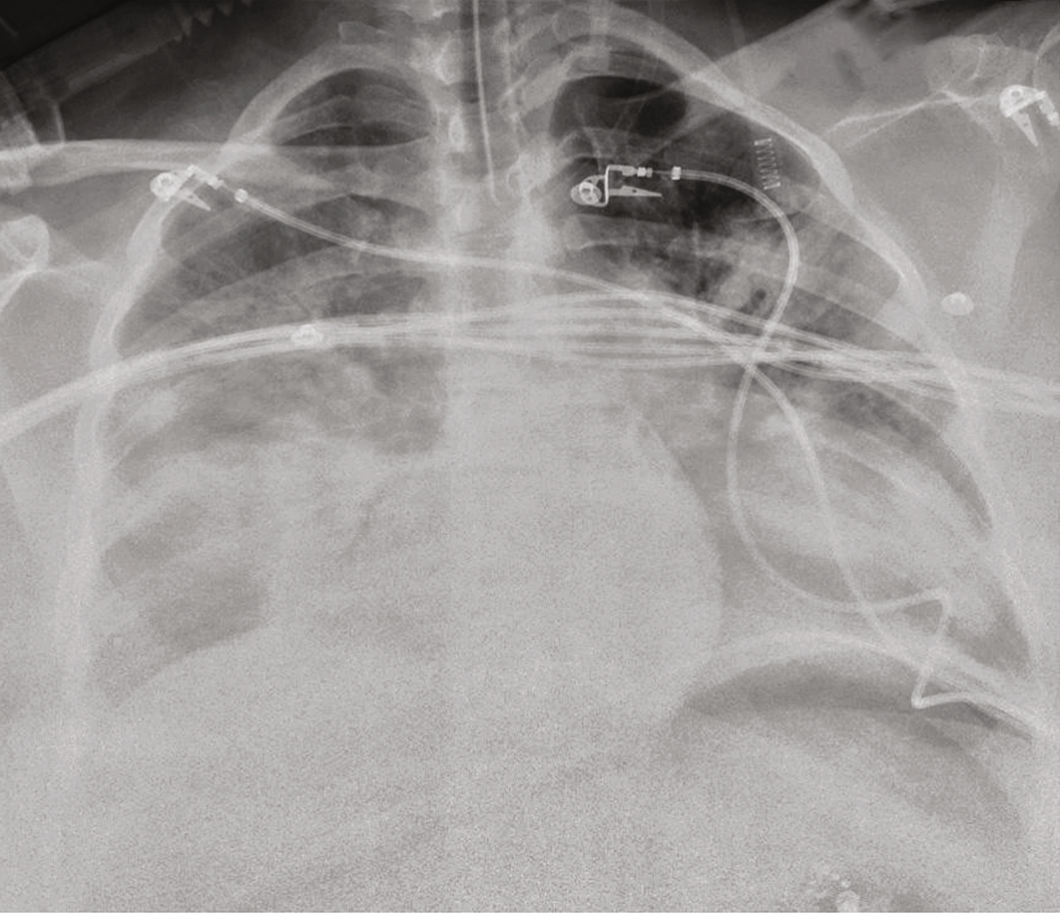
Chest radiograph shows interval worsening of bilateral lower lobe predominant diffuse reticular and alveolar airspace opacifications with trace bilateral pleural effusions.

Maternal-fetal medicine (MFM), obstetric, neonatal, and medical intensive care teams were alerted prior to arrival. Patient arrived to our MICU sedated and intubated. She was initially requiring vecuronium pushes to achieve vent synchrony and oxygen saturation above 92%. Chest radiograph did show worsening of lower lobe predominant diffuse reticular and alveolar airspace opacifications (Figure 5). Mechanical ventilation settings were modified to TV of 460 ml (6 ml/kg), RR of 24, PEEP of 12, and 100% FiO2. Arterial blood gas (ABG) on this setting includes a pH of 7.19, PaCO2 of 63 mmHg, and PaO2 of 80 mmHg. The computed PaO2 : FiO2 ratio was 80 indicative of severe acute respiratory distress syndrome (ARDS). The respiratory rate was increased to 28/minute. On exam, her membranes had spontaneously ruptured, and her cervix was dilated to 4 cm and 50% effaced. Bedside ultrasound showed decreased amniotic fluid index and cephalic presentation. The fetal heart rate was 135 beats per minutes (bpm) with overall minimal variability and positive acceleration response on scalp stimulation. Induction of labor with IV oxytocin was started with initial plan to attempt vaginal delivery with the assisted second stage. However, plans for emergency cesarean delivery were in place in the event it was indicated. IV heparin was continued. This plan was reviewed in the multidisciplinary approach including the medical and neonatal intensive care, obstetric, MFM, and anesthesiology teams.

Eight hours after admission, patient was saturating in the high 88-91% range on a PEEP of 12 and 100% FiO2. ABG on this setting showed a pH of 7.21, PaCO2 of 55 mmHg, and PaO2 of 82 mmHg. Plateau pressure was high, ranging between 38 and 40 cm H20.This was partially due to patient's body habitus, morbid obesity, and a gravid uterus. The severe hypoxemia was deemed potentially harmful to the fetus, and various options were considered to improve patient's oxygenation.

Given her gravid uterus, active labor, and BMI >50 kg/m^2^, prone ventilation was not an option. As a rescue measure, PEEP was then increased to 16 despite the high plateau pressure and RR to 32/minute. She received additional doses of paralytics to maintain vent synchrony. IV heparin was held in preparation for imminent delivery. Given the mother's severe hypoxia and potential for deterioration due to fluid shifts during delivery, extracorporeal membrane oxygenation (ECMO) was also discussed. An emergent discussion was held with another tertiary care hospital within the city with ECMO expertise. The patient was accepted there, in case she required ECMO, and our cardiothoracic surgeon was on board to establish femoral and jugular access in anticipation of emergent ECMO needs in the operating room, with improvement of oxygen status and respiratory acidosis, respectively, as evidenced by pH of 7.31, PaCO2 43 mmHg, and PaO2 377 mmHg on repeat ABG. FiO2 was then decreased to 90%.

Cervical dilatation remained relatively unchanged over the next hour or so, with three contractions palpated to be occurring within 10 minutes. Fetal heart monitoring showed minimal variability with no acceleration response on scalp stimulation. In view of this nonreassuring fetal heart tracing, the decision was made to proceed with cesarean delivery in the operating room. The medical intensive care, obstetric, MFM, neonatal intensive care, anesthesiology, cardiothoracic surgery teams were in coordination to ensure the stability of the patient and to address anticipated fetomaternal complications. Patient remained stable throughout the cesarean section and did not require ECMO.

She gave birth to an early term male infant with APGAR scores of 1 at 1^st^ minute, 2 at 5^th^ minute, and 4 at 10^th^ until the 20^th^ minute. As anticipated prior to delivery, the neonate required invasive mechanical ventilation, due to prolonged exposure to sedatives and paralytics from the mother. He needed bag mask ventilation with 100% FiO2 without return of spontaneous breathing. The heart rate was less than 60 beats per minute (bpm) requiring 30-second chest compression. After 5 minutes, the heart rate was 120 bpm; however, he was hypoxic to 85% while on 100% FiO2 necessitating mechanical ventilation. Neonate was then admitted to neonatal ICU which was tested for SARS-CoV2 which turned out negative twice. The following day, patient's baby was extubated to room air and was subsequently released on his 3^rd^ day of life to his aunt for primary care as his father was still positive for SARS-COV2.

In the interim, patient received a five-day course vancomycin and ceftriaxone for sputum culture growing *Haemophilus influenzae* and methicillin-resistant *Staphyloccus aureus*. There was a very low suspicion for bacterial superinfection given transient fever, mild leukocytosis with lymphocytic predominance, and absence of consolidation on chest radiograph. Most of the treatment were geared towards supportive management for COVID-19 and acute respiratory distress syndrome.

On the other hand, patient remained intubated, with decreasing oxygen requirements in the postpartum period. Her hypoxia improved significantly after delivery, despite a newly diagnosed cardiomyopathy as evidenced with 45% ejection fraction on echocardiography. IV heparin was resumed 6 hours postpartum. She was eventually extubated to bilevel positive airway pressure (BiPAP) on the 6^th^ day of intubation. She developed postextubation stridor necessitating administration of racemic epinephrine and solumedrol. She finished the 10-day course of remdesivir and 5-day course of IV methylprednisolone. IV heparin was also transitioned to subcutaneous enoxaparin. Patient was discharged to acute inpatient rehabilitation facility after 19 days of hospitalization.

## 3. Discussion

**Figure 6:**
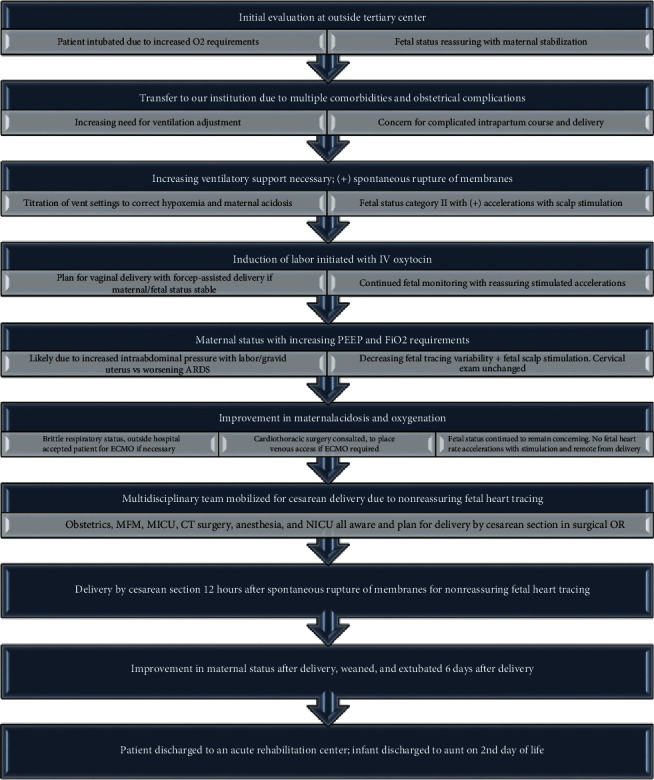
Summary of multidisciplinary management and outcomes of our COVID-19 pregnant patient.

This case highlights the clinical course of the COVID-19 infection in a pregnant woman with multiple comorbidities, the importance of taking into consideration physiologic maternal adaptations, and the intricate critical care aspects in managing the patient with anticipated fetomaternal complications from COVID-19-related ARDS and as well as the multidisciplinary approach to optimize maternal and neonatal outcomes (Figure 6).

Patient's current pregnancy with her comorbidities including malignancy, asthma ,and morbid obesity further complicated by pulmonary embolism during her hospitalization may have largely contributed to the development of severe acute respiratory distress syndrome (ARDS) in the setting of COVID-19 pneumonia [7–11].

Many potential challenges confronted the medical and neonatal intensive care, obstetric, and maternal fetal medicine teams in managing patient's severe hypoxemia with severe ARDS. It is of paramount importance to take into consideration maternal physiologic adaptations to pregnancy. Apart from leaving the woman more vulnerable to cell-mediated viral infection such as COVID-19, pregnant women are more susceptible to rapid cardio-pulmonary decompensation due to reduced cardiac and pulmonary reserves [12]. Particularly in the third trimester, the gravid uterus decreases functional residual capacity and expiratory reserve volume, which can potentially increase the risk of severe hypoxemia especially those who are critically ill [13]. Moreover, the physiologic adaptations to labor, delivery, and the immediate postpartum should also be considered as these could exacerbate the dysregulated inflammatory cascade in the setting of an underlying severe systemic infection. These physiologic changes include significant fluid shifts between the interstitial, intracellular, and intravascular compartments, maximization of the maternal cardiac output, autotransfusion of up to 500 mL of blood back into the intravascular compartment, catecholamine surge, and release of inflammatory mediators within the endothelium [11]. These in the setting of COVID-19 infection could place the patient at a higher risk for developing endothelial dysfunction, pulmonary edema, myocardial edema, and cardiac dysfunction [14]. Such considerations were at the forefront, in the minds of the entire medical and surgical team in optimizing patient's oxygenation while she was mechanically ventilated before, during and after the delivery.

While patient's labor was induced with IV oxytocin, options to improve patient's oxygenation prior to the contemplated delivery were maximized. First, ventilatory goals include consideration for the diminished functional residual capacity, higher PEEP requirement, greater physiologic tidal volume, and less lung compliance from higher innate plateau pressures due to diaphragmatic compression by the gravid uterus and rotund abdomen and chest wall compression from enlarged breast tissue [12, 15]. These presented a challenge to the “lung protective” strategy for mechanical ventilation in pregnant patients. It was then reasonable to increase the PEEP and FiO2 of this patient to meet oxygenation target. Second is the use of neuromuscular blocking agents with sedation in the early phase of ARDS. It was found to have added clinical benefit by minimizing the manifestations of ventilator-induced lung injury by reducing pulmonary and systemic production of inflammatory mediators [14]. Patient did receive *cis*-atracurium drip in another hospital before transfer and intermittent vecuronium pushes while admitted to our ICU. Patient ended up with cesarean delivery at term for nonreassuring fetal heart status with stable maternal status after correcting respiratory acidosis and achieving oxygenation goals. This is congruous with the result of a systematic review and meta-analysis showing increased cesarean delivery rate among pregnant women with COVID-19 [16].

Although the safety and efficacy of prone ventilation in pregnancy has been documented [17, 18], patient's weight, size and active labor precluded the performance of this maneuver. Pregnancy is also a contraindication for prone ventilation per our institutional policy. Thus, venovenous ECMO was considered as a life-saving salvage therapy in the event of worsening oxygenation and hemodynamic compromise during and after the delivery [19]. Cardiothoracic surgeon placed central venous access in the internal jugular and femoral veins prior to the cesarean delivery, in anticipation of ECMO. In the interim, patient was already accepted in another tertiary hospital for ECMO should the need arise.

As most of the trials for treatment of COVID-19 exclude pregnant women, few agents although available remain investigational in terms of their clinical utility. These include remdesivir, systemic steroids, and convalescent plasma which are offered in different hospitals under compassionate-use protocol [20–22]. After a lengthy discussion, patient and her husband provided consent for the administration of the remdesivir and convalescent plasma early in the course of patient's hospitalization. Timely initiation of bronchodilator and steroid therapy, as well as IV heparin for asthma exacerbation and pulmonary embolism, respectively, might have afforded additional benefit leading to patient's excellent overall clinical outcomes.

As to the neonatal outcome, patient's baby improved clinically after resuscitation and mechanical ventilation for 24 hours. Cardiorespiratory depression was likely secondary to prolonged maternal exposure to sedation and paralytics. The SARS-CoV-2 PCR test of the baby turned out negative which is consistent with recent findings documenting no evidence of vertical transmission in women with COVID-19 pneumonia [6, 23].

**Figure 7:**
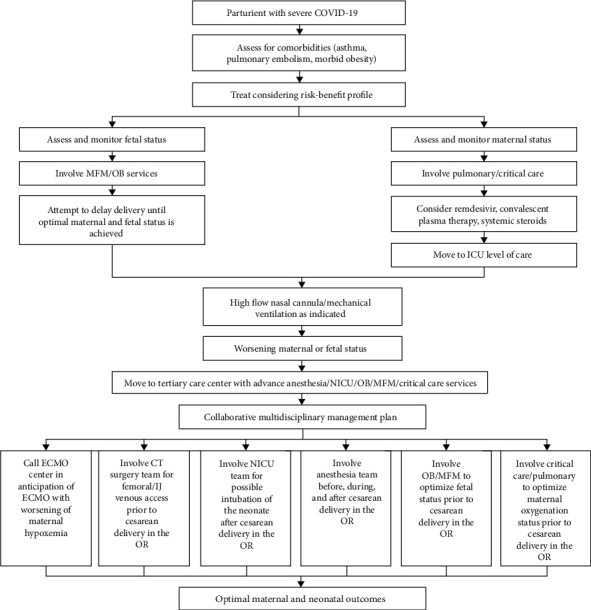
Proposed algorithm for multidisciplinary management of a parturient with severe COVID-19.

A proposed algorithmic approach to the management of a parturient with severe COVID-19 is included in this report (Figure 7). This entailed valuable input from multiple disciplines involving the medical and neonatal intensive care, obstetric, MFM, anesthesiology, and cardiothoracic surgery teams. A framework utilizing direct open communication that optimized team dynamics was established, along with active involvement of patient's husband in the decision-making vis-à-vis available therapeutic options. Maternal and neonatal outcomes were excellent as a result of highly coordinated care through a multidisciplinary approach.

## Data Availability

The data used to support the findings of this study are included within the article.
